# Detection of polyol accumulation in a new ovarian carcinoma cell line, CABA I: a^1^H NMR study

**DOI:** 10.1038/sj.bjc.6600189

**Published:** 2002-04-08

**Authors:** A Ferretti, S D'Ascenzo, A Knijn, E Iorio, V Dolo, A Pavan, F Podo

**Affiliations:** Laboratory of Cell Biology, Istituto Superiore di Sanità, Viale Regina Elena 299, 00161 Roma, Italy; Department of Experimental Medicine, University of L'Aquila, Via Vetoio-Coppito 2, 67100 L'Aquila, Italy

**Keywords:** ovarian cancer, NMR, sorbitol, *myo-*inositol, glutathione

## Abstract

Ovarian carcinomas represent a major form of gynaecological malignancies, whose treatment consists mainly of surgery and chemotherapy. Besides the difficulty of prognosis, therapy of ovarian carcinomas has reached scarce improvement, as a consequence of lack of efficacy and development of drug-resistance. The need of different biochemical and functional parameters has grown, in order to obtain a larger view on processes of biological and clinical significance. In this paper we report novel metabolic features detected in a series of different human ovary carcinoma lines, by ^1^H NMR spectroscopy of intact cells and their extracts. Most importantly, a new ovarian adenocarcinoma line CABA I, showed strong signals in the spectral region between 3.5 and 4.0 p.p.m., assigned for the first time to the polyol sorbitol (39±11 nmol/10^6^ cells). ^13^C NMR analyses of these cells incubated with [1-^13^C]-D-glucose demonstrated labelled-sorbitol formation. The other ovarian carcinoma cell lines (OVCAR-3, IGROV 1, SK-OV-3 and OVCA432), showed, in the same spectral region, intense resonances from other metabolites: glutathione (up to 30 nmol/10^6^ cells) and *myo*-inositol (up to 50 nmol/10^6^ cells). Biochemical and biological functions are suggested for these compounds in human ovarian carcinoma cells, especially in relation to their possible role in cell detoxification mechanisms during tumour progression.

*British Journal of Cancer* (2002) **86**, 1180–1187. DOI: 10.1038/sj/bjc/6600189 www.bjcancer.com

© 2002 Cancer Research UK

## 

Human epithelial ovarian tumours represent a major type of gynaecologic malignancy. The vast majority of ovarian carcinomas arise as a result of malignant transformation of the ovarian surface epithelium. Because of the invasive nature of these tumours and the current inability to detect the disease at early stages (Stage I or II), a significant number of women are initially diagnosed only after the neoplasia has already spread throughout the peritoneal cavity (Stage III or IV). The triggering event(s) in the generation and development of ovarian cancer are not yet well understood. Propagation of epithelial ovarian cancer occurs essentially as a direct infiltration into the peritoneal cavity, upon exfoliation of cells from the primary tumour and dissemination by the peritoneal fluid, with subsequent implantation, invasion and growth (Williams, 2000). To enable the development of appropriate screening strategies for ovarian cancer, the processes of carcinogenesis and tumour progression need to be understood. Since little is as yet known about the morphological and molecular steps involved in the initiation and progression of epithelial ovarian cancer, new biochemical and physiological information, as well as measurement of functional parameters, are of extreme importance in order to obtain a more detailed clinical picture of these tumours.

By allowing non-invasive monitoring of biochemical pathways in intact cells and tissues and their modulations under particular pathological conditions, NMR spectroscopy offers novel approaches to detect metabolic alterations associated with malignant phenotypes of ovarian cancer cells *in vitro*, as a basis for a possible *in vivo* monitoring of clinical lesions. In particular, NMR spectra of intact cells and tissues allow detection and quantification of a number of intracellular metabolites (present at intracellular concentrations >about 0.5 mM) and their fluxes in either ubiquitous or tissue-specific biochemical pathways. Among these, particular attention has been devoted to metabolites involved in phospholipid biosynthesis and catabolism (reviewed in Podo, 1999), in oxidative and non-oxidative glucose consumption and in cell bioenergetics (Gadian, 1995; Magistretti *et al*, 1999), as well as to the production of neurotransmitters, neuroaminoacids and *myo-*inositol in brain (Danielsen and Ross, 1999; Ross, 2000) and accumulation of citrate in prostate (Kurhanewicz *et al*, 1996).

The detection by NMR of substrates and derivatives of these pathways and the measurement of their changes in concentration in tumour with respect to non-tumour cells, not only may allow relevant information on activation/inhibition of metabolic processes as they occur in cells, animal models and clinical lesions, but may also provide new biochemical markers of *in vivo* tumour progression and response to therapy. Examples of major ^1^H NMR spectral variations reported in tumours, with respect to normal cells and tissues, are a generally elevated intensity of choline-containing metabolites (‘Cho-peak’, 3.2 p.p.m.), mainly due to increased levels of phosphocholine (PCho) in brain, breast, prostate and other tumours (Negendank *et al*, 1996; Podo, 1999; Aboagye and Bhujwalla, 1999); loss of N-acetylaspartate, a putative neuroaminoacid, in gliomas (Ross, 2000); increase of *myo-*inositol in some brain tumours (Barba *et al*, 2001); and decrease of citrate in prostate carcinoma (Kurhanewicz *et al*, 1995). Furthermore, several tumour cells and tissue specimens exhibit ^1^H NMR signals attributed to either membrane or intracellular mobile lipid domains (ML), whose fatty chains are endowed with a high degree of mobility, not compatible with the anisotropic packing in the lamellar phase (Mountford *et al*, 1993; Callies *et al*, 1993). The relative intensity of these signals was found to discriminate pre-malignant from invasive cancer in tissue specimens dissected from human uterine cervix and thyroid follicular adenomas from carcinomas (Mountford *et al*, 1996). However, elevated ML levels are not exclusively associated with the malignant phenotype, since they were also induced by cell activation in lymphocytes and lymphoblasts (Veale *et al*, 1997) and were detected in some embryo-derived cells (May *et al*, 1986; Ferretti *et al*, 1999), as well as in different types of cells undergoing apoptosis (Blankenberg *et al*, 1997; Di Vito *et al*, 2001). Finally, a rather intense resonance profile may be observed in the so-called ‘CH’ or ‘sugar’ spectral region (3.5–4.0 p.p.m.) of some tumours (e.g. cervical intraepithelial neoplasias), whose individual contributions, however, have not yet been clearly identified (Mountford *et al*, 1993; Callies *et al*, 1993).

So far, only a few NMR studies have been reported on human ovarian pathologies, essentially confined to the analysis of fluids from ovarian cysts. By these analyses, significant differences in a variety of soluble metabolite concentrations (some still unassigned) were found between benign and malignant ovarian cysts (Massuger *et al*, 1998; Boss *et al*, 2000). No direct investigations were conducted on human ovarian adenocarcinomas.

This paper reports the results of a ^1^H NMR study on five human ovarian carcinoma cell lines of different origin. This is to our knowledge the first reported evidence on the presence of a polyol in an ovarian cancer cell line (CABA I). High levels of inositol and glutathione were instead detected in the other cell lines examined in this study. The results suggest the interest of further investigating the biochemical pathways responsible for alternative production and accumulation of these soluble metabolites and their implications in self-detoxification processes in human ovarian cancer cell lines.

## MATERIALS AND METHODS

### Cell lines

The characteristics and origin of all cell lines used in this study are summarised in Table 1

**Table 1:**
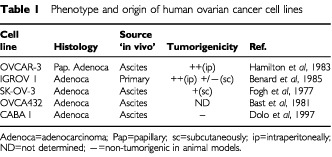
Phenotype and origin of human ovarian cancer cell lines

(where they are listed in order of decreasing *in vivo* tumorigenicity in animal models). The CABA I cell line was established from the ascitic fluid of a patient with ovarian carcinoma prior to any drug treatment. The cell line exhibits complex cytogenetic and mutation patterns, with the possible deletion of the entire exon 5 of the p53 gene (Dolo *et al*, 1997). SK-OV-3, OVCA432, IGROV 1 and OVCAR-3 cell lines were kindly provided by Dr S Canevari (Istituto Nazionale Tumori, Milano, Italy). Cells were grown as monolayers in RPMI 1640 (Euroclone, Devon, UK) with 10% foetal calf serum (FCS, Euroclone). For each experiment, monolayer cells were harvested in 0.05% Trypsin and 0.02% EDTA (Euroclone), resuspended in RPMI/FCS (complete medium) and then washed three times in PBS. The cells were counted and their viability (80–90%) and membrane integrity assessed by Trypan blue (Euroclone) dye exclusion, both before and after NMR measurements, (during which there was no significant drop in cell viability). All cell lines were periodically tested for mycoplasma contamination.

### NMR spectroscopy

Intact cells were resuspended in 600 μl of PBS in 70% (v/v) D_2_O (pH=7.3) and transferred into 5 mm NMR tubes. The ^1^H NMR experiments on intact cells were generally carried out on a Bruker Avance 400 MHz WB (9.4 T) spectrometer, at 25°C. Some spectral quantification was also performed on measurements recorded at 200 MHz, using an analytical Varian Gemini 200 NMR spectrometer. One-dimensional (1D) analyses were carried out using a single-pulse (60°) sequence, preceded by 1.0 s presaturation for water signal suppression (spectral width 10.013 p.p.m.), the total measurement time being 17 min for 320 scans. Two-dimensional (2D) ^1^H-NMR homonuclear shift correlation (COSY) experiments were performed using gradient pulses for selection; the COSY spectra were acquired with eight transients, 256 time domain points in *t*_1_, acquisition time 0.27 s, spectral width of 4006.41 Hz in both dimensions, repetition time of 1 s.

Experiments on ethanolic extracts of cells were carried out applying the same pulse sequence as before, at the equilibrium of magnetisation (90° pulses and 30.0 s interpulse delay time). ^13^C NMR analyses were performed on intact cells incubated with [1-^13^C]-D-glucose (Merck Sharp and Dohme, Canada, 99.1% isotopic substitution) as well as on their extracts, utilising a sequence with power-gated decoupling pulses. The ethanolic extracts were also analysed by 2D ^1^H/^13^C correlation spectroscopy via heteronuclear zero and double quantum coherence, using the HETCOR-inv4tp sequence (Bax *et al*, 1983) or the modified inv4gp version, that utilises gradient pulses for selection; the samples were recorded in the proton-detected mode.

All types of 1D and 2D NMR analyses were repeated on standard compounds (sorbitol, glutathione, *myo-*inositol; Sigma-Aldrich, Milano, Italy) for both verification of signal assignments and peak area quantification.

Quantitative data analysis of spectra of intact cells and their extracts was performed in the frequency domain using the Bruker Win-NMR software package. Free induction decays were zero-filled to 32 k data points and Fourier-transformed, after which base line correction was performed, applying a cubic splines model function through appropriate data points. Quantitation was then obtained either through integration (cell extracts) or deconvolution (intact cells) of resonance peaks.

The concentration values of water-soluble metabolites were calculated by peak integration in the spectra of ethanolic cell extracts. In particular, for the quantification of sorbitol all signals were utilised, with reference to a standard solution of this compound (5 mM); the ‘doublet of doublets’ centred at about 3.0 p.p.m., due to the CH_2_ group of cysteine was used for quantifying glutathione, while the signals around 3.5 p.p.m. (H1 and H3) were used for *myo-*inositol.

### Ethanolic cell extracts

At the end of NMR experiments, cells were extracted by adding five volumes of ethanolic solution (EtOH:H_2_O, 70:30 v/v). The samples were sonicated at 20 kHz by a MSE ultrasonic disintegrator Mk2 (exponential probe, 8 μm peak to peak) and centrifuged at 14 000×**g** for 30 min. The supernatants were lyophilised two times in a RVT 4104 Savant lyophiliser, and the residue resuspended in 700 μl D_2_O (Sigma-Aldrich, Milano, Italy) containing 3-trimethylsilylpropionate-2,2,3,3-D4 0.1 mM as internal standard (Merck & Co., Montreal, Canada).

## RESULTS

### Soluble metabolites

**Figure 1:**
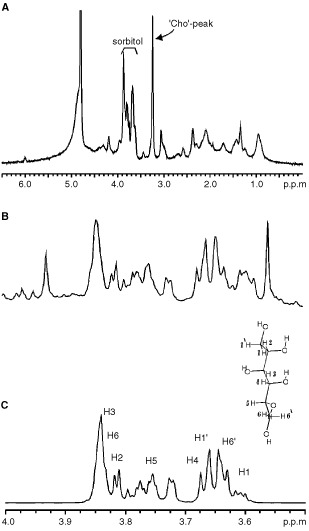
(**A**) ^1^H NMR spectrum (400.1 MHz) of intact human ovarian carcinoma cells CABA I, with assignment of signals due to N^+^(CH_3_)_3_ from choline-containing compounds (‘Cho’-peak) and sorbitol; (**B**) expanded spectral ‘CH-region’ in CABA I cell extract; (**C**) sorbitol standard solution.

^1^H NMR spectra of intact CABA I cancer cells are shown in Figures 1A

and 2

**Figure 2:**
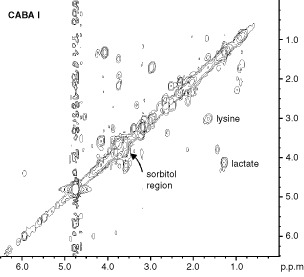
2D ^1^H COSY experiment on intact CABA I cells. Some relevant cross-peak assignments are indicated. For further details see text.

. The strong resonances dominating the so-called ‘CH-region’ between 3.5 and 4.0 p.p.m. (Figure 1A) were attributed to sorbitol. The assignment was based upon analytical comparison of the 1D spectrum of the ethanolic cell extract (Figure 1B) with that of a standard solution of D-sorbitol (Figure 1C) and confirmed by 2D-COSY experiments (Figure 2).

**Scheme 1:**
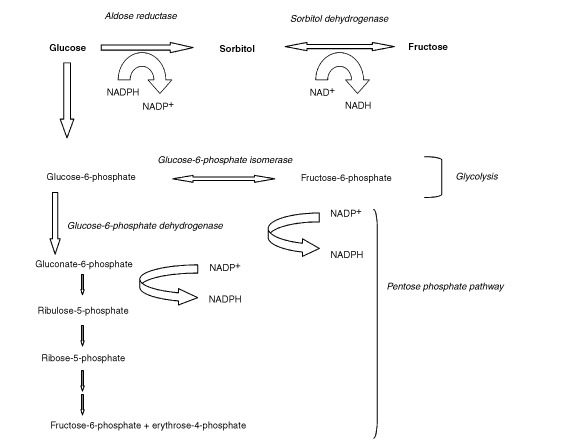
Biochemical reactions of glucose metabolising pathways

**Figure 3:**
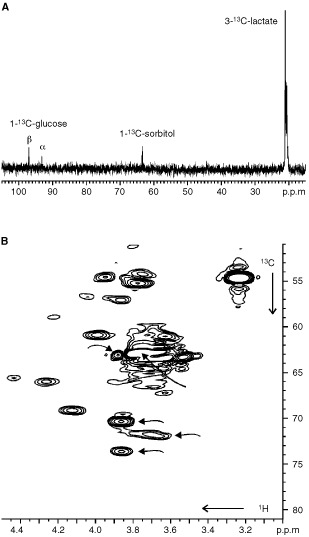
(**A**) ^13^C NMR spectrum (100.6 MHz) of ethanolic extract of CABA I cells, following incubation with [1-^13^C]-D-glucose; (**B**) 2D^ 1^H/^13^C HETCOR analysis of the same sample (expanded regions); the arrows indicate ^1^H/^13^C cross-peaks from sorbitol.

It is reasonable to propose that accumulation of sorbitol in these cells is mainly dependent upon the activity of aldose reductase, an enzyme utilising glucose as substrate, with the simultaneous oxidation of NADPH (Scheme 1

). In order to verify this hypothesis, CABA I cells, harvested and collected at 72 h of culture, were incubated in the presence of 20 mM [1-^13^C]-D-glucose in a 5 mm NMR tube at 25°C, and NMR analyses performed on intact and viable cells at various time intervals, up to 10 h (data not shown). The formation of [1-^13^C]-sorbitol, concomitant with the decrease of the anomeric glucose carbons (both the α and β isoforms, respectively, at 93.2 and 96.9 p.p.m.) and increase in lactate (3-^13^C, 21.0 p.p.m.), was confirmed by analysing cell extracts by both 1D ^13^C NMR (signal at 63.4 p.p.m., Figure 3A

) and 2D ^13^C/^1^H HETCOR spectra (cross-peaks at 3.84/74.2 p.p.m., 3.67/72.3 p.p.m., 3.84/70.8 p.p.m., 3.86/63.8 p.p.m. and 3.80/63.4 p.p.m., Figure 3B). At the end of incubation, when 94% of glucose had been converted into other metabolites, sorbitol reached a concentration of 0.7 mM, indicating that, under these conditions, at least 3.7% of the substrate had been utilised to produce the polyol.

**Figure 4:**
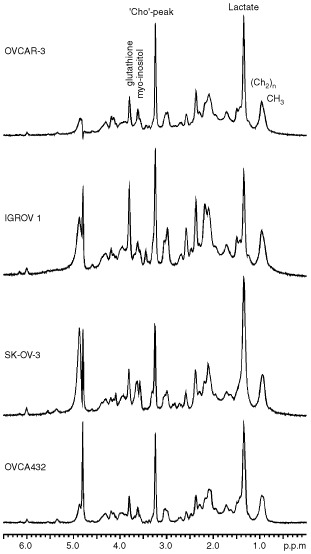
Representative ^1^H NMR spectra (400.1 MHz) of different intact human ovarian cancer cell lines with some peak assignments. N^+^(CH_3_)_3_ from choline-containing compounds (‘Cho’-peak); (CH_2_)_n_ and CH_3_ from mobile lipids.

On the other hand, no appreciable levels of sorbitol were detected in the spectral patterns of the other four ovary carcinoma cell lines under investigation (Figure 4

). The spectra of these cells were characterised, in the 3.5–4.0 p.p.m. region, by intense signals due to other metabolites, present at variable concentration levels.

**Figure 5:**
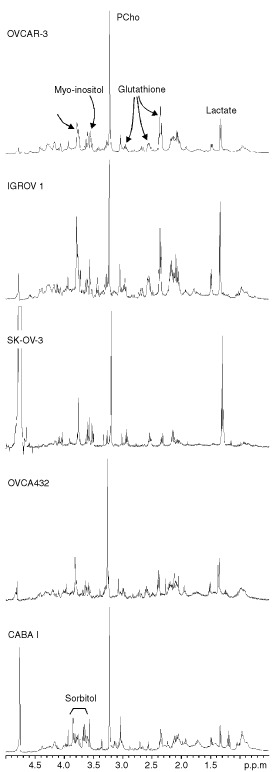
^1^H NMR spectra of ethanolic extracts of ovarian cancer cell lines. PCho=phosphocholine.

**Figure 6:**
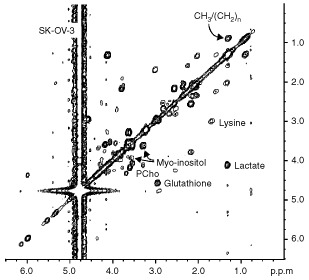
2D ^1^H COSY experiments on intact SK-OV-3 cells. Some relevant cross-peak assignments are indicated. For further details see text.

Analysis of aqueous cell extracts (Figure 5

) demonstrated that the most relevant peak, observed at 3.79 p.p.m., was due to the glycine residue of glutathione, whose other signals were detected at 2.2, 2.5, 3.0 and 4.5 p.p.m. In particular, the presence of reduced glutathione was indicated by the cross-peak at 3.0/4.5 p.p.m. in the 2D-COSY spectra, as can be seen for example in Figure 5 for SK-OV-3 intact cells. *Myo-*inositol was detected in the spectra of all these cells, as demonstrated by its characteristic signals centred at 3.52 p.p.m. (due to H1 and H3) and the typical cross-peaks at 3.5 p.p.m./4.00 p.p.m. in the 1D and 2D-COSY experiments of intact cells, respectively (Figures 4 and 6

). Likewise, all multiplets of *myo*-inositol, respectively due to H5 (3.26 p.p.m.), H4 (3.61 p.p.m.) and H2 (4.00 p.p.m.) were clearly resolved in the spectra of cell extracts (Figure 5).

The doublet at 1.33 p.p.m. due to H3 from lactate (Figure 4) showed a large variability between experiments of intact cells with no significant differences between the cell lines (the peak area ratio of the lactate doublet to the lysine signal at 1.7 p.p.m. (H3 and H5) was of 0.31±0.12 in CABA I (five experiments) and 0.89±0.75 in the other four ovarian cell lines (in total nine experiments).

Table 2

**Table 2:**
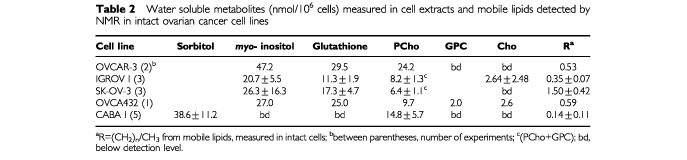
Water soluble metabolites (nmol/10^6^ cells) measured in cell extracts and mobile lipids detected by NMR in intact ovarian cancer cell lines

reports the concentrations of the most relevant water-soluble metabolites measured by ^1^H NMR in the analysed ovarian cancer cells. The values were determined by peak integration in the spectra of ethanolic cell extracts. These analyses confirmed high levels of sorbitol only in CABA I cells, while glutathione and *myo-*inositol (but not sorbitol) were present in the other cell lines. The concentration value of 17.3±4.7 nmol/10^6^ cells, corresponding to 75.2±20.0 nmol/mg protein, measured for glutathione in SK-OV-3, was in good agreement with that previously reported by Hosking *et al*, 1990, for the same cell line (80.7±18.0 nmol/mg protein).

^1^H NMR analyses of cell extracts also allowed quantification of the molecular components contributing to the so-called ‘Cho-peak’ (3.2 p.p.m. in the spectra of intact cells), mainly PCho (3.22 p.p.m.) and, to much lower extents, glycerophosphorylcholine (GPC, 3.23 p.p.m.) and free choline (Cho, 3.20 p.p.m.). The concentration of PCho reached a mean value of 24 nmol/10^6^ cells in the most tumorigenic cell line (OVCAR-3) and about 15 nmol/10^6^ cells in CABA I (Table 2).

### Mobile lipids

The proton spectra of some intact ovarian cancer cells showed the typical signals arising from ‘mobile lipids’, i.e. lipids comprised in structures endowed of sufficiently high isotropic mobility to be detected by high resolution NMR spectroscopy. In particular, the presence of ML was recognised by the peak at 1.27 p.p.m., due to (CH_2_)_n_ segments of fatty acyl chains, next to the peak of lactate at 1.33 p.p.m., and by the large composite resonance centred at 0.89 p.p.m., typically comprising contributions from the chains' terminal CH_3_, superimposed on those of cholesterol methyl groups (at position 18, 19, 21, 26, 27) and of amino acids' methyl groups (Figure 4). The ratio (R) between the 1.27 p.p.m. and the 0.89 p.p.m. peak areas, usually adopted as empirical parameter for relative ML quantification, was 1.5±0.4 in SK-OV-3 cells, in which the presence of ML was confirmed by the typical cross peak at 0.9 p.p.m./1.3 p.p.m. (Figure 6). The other ovarian cancer cell lines exhibited much lower R values (Table 2), while CABA I cells were practically deprived of mobile (CH_2_)_n_ segments, as also shown by 2D COSY spectra (Figure 2).

## DISCUSSION

This study provides evidence on the presence, in five human ovarian carcinoma cell lines, of ^1^H NMR-detectable amounts of metabolites such as sorbitol, reduced glutathione and *myo-*inositol. These compounds are typically implicated in cellular detoxification pathways, although they may act as osmolites. Sorbitol may furthermore compete for intracellular stores of *myo-*inositol, inducing depletion of this metabolite (Kuruvilla and Eichberg, 1998). As a consequence, different levels of *myo-*inositol may also influence the biosynthesis and turnover of phospholipids.

CABA I cells were characterised by high levels of sorbitol (39±11 nmol/10^6^ cells). Accumulation of this compound has been reported to occur in some non-tumour tissues, such as the crystalline lens and nerves of patients affected by diabetes (Kuruvilla and Eichberg, 1998), as a complication of this pathology (Kinoshita, 1990). A high level of sorbitol may even induce fracture of the lens. Regarding tumour cells, an elevated concentration of sorbitol has been found to induce resistance to cis-platinum in human non-small-cell lung cancer cell lines, by modulating the activity of Na^+^, K^+^ ATPase (Bando *et al*, 1997).

Sorbitol is mainly produced in the cells from glucose by the aldose reductase pathway (Scheme 1). The general role of such enzyme, expressed in some tissues and organs, is not yet well clarified. Regarding tumours, an aldose reductase activity has been identified in rat hepatoma, in which it was suggested to play an important role in cell detoxification from harmful metabolites, such as aldehydes, generated by intracellular metabolism. Moreover, this enzyme has been reported to display a high sequence homology with a novel human aldose reductase, overexpressed in human liver cancer (Cao *et al*, 1998).

Recent studies also reported that aldose reductase can convert daunorubicin into its reduced form, daunorubicinol, thus decreasing the pharmacological activity of this anti-tumour drug (Ax *et al*, 2000).

This body of evidence suggests that accumulation of sorbitol in CABA I cells might be an index of increased metabolic flux through the aldose reductase pathway, by which these fast growing cancer cells would likely enhance their capability of self-detoxification, through reduction of aldehydes or other similar (either endogenous or exogenous) compounds, including anti-cancer drugs.

Additionally, an indirect detoxification process could be triggered in these cells, by activation of the pentose phosphate shunt, in which NADP produced from NADPH in the aldose reductase pathway is effectively utilised. Besides, the pentose phosphate shunt is directly involved in nucleic acid ribose synthesis and in proliferation of pancreatic and lung epithelial carcinoma cells; the control of this shunt may be critical in cancer treatment, as recently reported by Boros *et al*, 2000. Furthermore, sorbitol could as well be synthesised from fructose via the activated pentose phosphate pathway from glucose and ribose and sorbitol dehydrogenase (Jans *et al*, 1989).

Under our experimental conditions, we could directly demonstrate the formation of ^13^C-labelled sorbitol from [1-^13^C]glucose in CABA I cells, thus confirming that this polyol can effectively be synthetised from this common substrate, through the described fluxes.

Quantification of the individual contributions provided by these detoxification pathways to the elevated concentration of sorbitol, and their alterations under different conditions of cell exposure to either cytotoxic drugs and/or to supplementation with specific substrates (such as folate, reported to interfere with the activity of sorbitol dehydrogenase (Vandenberghe *et al*, 1995)) would enhance our understanding of the significance of sorbitol accumulation in relation to the responsiveness of CABA I cells to combined anticancer therapies. This perspective appears particularly interesting in view of the recent demonstration that CABA I cells possess mutated α-folate receptors, associated with molecules regulating cell proliferation, but with impaired affinity for folates (Mangiarotti *et al*, 2001).

The other four carcinoma cell lines exhibited, instead of sorbitol, high levels of glutathione and *myo-*inositol, metabolites likewise known for being involved in detoxification processes of the cells. In particular, the role of the glutathione system in the development and maintenance of multi-drug resistance has been demonstrated in some tumour cells (Hosking *et al*, 1990; Ferretti *et al*, 1993). Regarding *myo-*inositol, although the complexity of the pathways responsible for the synthesis and turnover of this metabolite so far prevented a clear elucidation of the role of this compound in cerebral tumours and in some cognitive diseases, the view is growing that *myo-*inositol does not act as a simple osmolyte (Ross, 2000).

The detection in the present work of high levels of *myo-*inositol and glutathione in ovarian cancer cells (in particular in the most tumorigenic line investigated, i.e. OVCAR-3) stimulates the interest of further investigating their role as cell detoxification agents and as possible indicators of tumour progression of ovarian cancer *in vivo*.

Besides identifying compounds, which mainly affect the CH region, ^1^H NMR allowed likewise the detection in intact ovarian cancer cells of a strong ‘Cho’–peak, mostly due to PCho. This metabolite reached substantial levels in some of the investigated cells, similar to and even higher than those detected in some cell lines derived from other human epithelial tumours, such as breast (Aboagye and Bhujwalla, 1999) or prostate (Ackerstaff *et al*, 2001) carcinomas.

Regarding mobile lipid domains, there was quite a large variability in the detection of their typical signals in ^1^H NMR spectra of the different intact carcinoma cell lines analysed in this study, with no association with their respective origin and/or *in vivo* tumorigenicity. Very similar R values were found in three cell lines IGROV 1, OVCAR-3 and OVCA432 (R∼0.5) which, differently from SK-OV-3 (R∼1.5) are characterised by high levels of α-folate receptors and by low or absent levels of caveolin-1 expression (Bagnoli *et al*, 2000). The existence of a possible relationship between the detection of NMR-visible ML domains and caveolin-1 expression deserves further investigation.

In conclusion, in this study we report the possibility to detect and quantify by ^1^H NMR previously unidentified components of intact ovarian carcinoma cell lines. This evidence may open novel ways to the analysis and interpretation of ^1^H NMR spectra of ovarian tumour tissues *in vivo* and *ex vivo*.
